# Effects of Operating Conditions and Pit Area Ratio on the Coefficient of Friction of Textured Assemblies in Lubricated Reciprocating Sliding

**DOI:** 10.3390/ma15207199

**Published:** 2022-10-15

**Authors:** Slawomir Wos, Waldemar Koszela, Andrzej Dzierwa, Pawel Pawlus

**Affiliations:** Department of Manufacturing Processes and Production Engineering, Rzeszów University of Technology, 35-959 Rzeszów, Poland

**Keywords:** surface texturing, friction, temperature, normal load, frequency of oscillation

## Abstract

The experiment was carried out in a reciprocating lubricated conformal sliding contact between steel discs of the same hardness. The effects of disc surface texturing on the friction coefficient at various operating conditions (temperature, normal load, and frequency of oscillations) were studied. Under various conditions, surface texturing caused friction reductions of sliding pairs. The largest reduction was 4.6 times at a lower temperature and 2.5 times at a higher temperature. The effect of the pit area ratio on the friction reduction was visible at a higher temperature. The highest dimple density of 25% corresponded to a lower coefficient of friction than the smallest density of 9%. The sliding pair with a dimple density of 17% led to large variation of the friction force. At lower temperatures, the coefficients of friction were lower compared to tests at higher temperatures.

## 1. Introduction

Surface topography greatly affects the properties related to contact mechanics [1,2]. However, it also influences friction and wear. Creation of dimples or grooves on co-acted surfaces, called surface texturing, can lead to improvements in the tribological properties of machined elements. Surface texturing is a way to increase the frictional, wear, and seizure resistances of sliding pairs. Textured surfaces contain dimples (cavities or oil pockets) or grooves created by various manufacturing processes. Spherical dimples are typically created. They are characterized by depths, diameters, and pit area ratios (densities of dimples). The roughness height of the areas without dimples is also important. The pit area ratio seems to be the most important parameter of textured surfaces [3] and is typically between 5 and 25% [4]. Two-process cylinder liners with micro grooves are examples of textured surfaces [5]. They can be created by plateau honing or laser honing [6].

However, the influence of the presence of dimples depends on the operating conditions and type of contact [7]. Results of wear patterns and wear particles can provide insight into how surface texture affects friction [8]. The effects of surface texturing in mixed lubrication are greater than those in the fluid lubrication regime [9]. The beneficial effects of surface texturing have been shown mainly under conformal contact conditions [10].

Oil pockets performing well at high speed and small load conditions (generation of hydrodynamic pressure) lead to small reductions in the coefficient of friction under low speed and high load regimes (lubricant retention ability) [3]. According to Hsu et al. [11] wide and shallow dimples are effective for hydrodynamic lubrication, while narrow and deep oil pockets reduce mixed or boundary friction. Therefore, the combination of large dimples effective for generating hydrodynamic lubrication and small dimples effective for improving running-in is a good choice [11,12]. Segu et al. [13,14] created multi-scale textured samples for tribological purposes, obtaining positive effects in dry and lubricated friction conditions. Grützmacher et al. achieved beneficial effects from multi-scale texturing of the shaft of journal bearings with mixed and hydrodynamic lubrications [15,16]. The authors in [17] presented a review of multi-scale surface texturing.

Typically, studies on the functional properties of textured surfaces are carried out under conditions of unidirectional sliding. In reciprocating conditions, the situation is complicated because of various conditions of work at various places of the stroke. The viscosity and additives of the lubricant can be a factor in different lubrication regimes during an oscillation. In addition, increasingly, lubricants use nanoparticles for high-temperature performance [18], which affect the tribological behaviors of textured surfaces. The co-action between piston rings and cylinder liners is the most popular example of the reciprocating contact of sliding surfaces. In the piston ring–cylinder liner assembly near top dead center, a decrease in piston velocity leads to a decrease in oil film thickness, and therefore mainly mixed or boundary friction occurs [19]. Surface texturing helps to increase the thickness of the oil film, leading to reduction of the asperity contact and decreased friction force [20,21]. Grabon et al. [22] found that the friction of the sliding pair consisting of the piston ring and honed cylinder liner decreased when the temperature decreased due to an increase in oil viscosity. Due to various operating conditions within one stroke, the density of oil pockets depend on the location on the cylinder liner [23]. Partial surface texturing of piston rings leads to fuel consumption reduction of about 4% [24,25].

Koszela et al. obtained a reduction in friction [26] and an increase in the effective power [27] of the internal combustion engine by introducing additional oil pockets created by burnishing the honed cylinder liner surface. The pit area ratio was 13%.

Vladescu et al. [28] found that under reciprocating motion in boundary lubrication (low speed), rectangular oil pockets should be deep, wide, and densely spaced. For higher speeds (transition from mixed to full-film lubrication), dimples should be narrow and sparsely spaced. They also found that under full lubrication, oil starvation occurred after reversals [29]. In [30], researchers found that decreases in friction and wear were proportional to the sum of volumes of rectangular oil pockets. Textured surfaces led to an increase in oil film thickness [31]. The distance between oil pockets on the surfaces of the cylinder liner depend on the reciprocating sliding speed. Analysis of the worn sample after reciprocating lubricated sliding showed surface damage at the end of the stroke and negligible wear at the central part of the stroke [32].

Researchers tested various oil pocket shapes in reciprocating sliding. Shen and Khonsari [33] optimized the shape of oil pockets in unidirectional and bidirectional sliding. They selected a trapezoid-like shape in bidirectional sliding. Morris et al. [34,35] analyzed the impacts of the presence of chevron-based oil pockets in reciprocating sliding. Lu et al. studied the tribological behaviors of square [36] and triangular [37] oil pockets on the flat surface in line contact during reciprocating lubricating sliding. Wos et al. [38] analyzed the effects of spherical oil pockets and oil pockets of a sandglass shape on friction reduction in reciprocating sliding. However, the circular shape of the dimples is the most widely used [6].

There are many methods of oil pocket creation. Burnishing [24,27,39,40] and abrasive jet machining [41,42] are promising techniques. However, laser texturing is the most widely used method.

One can see from this review of the literature that the effects of operating conditions on the friction of textured sliding pairs in lubricated reciprocating sliding have rarely been analyzed experimentally. Only Vladescu et al. [28] studied the effect of sliding speed on the optimal patterns of dimples. However, the effects of various operating parameters on the coefficient of friction of textured pairs in bidirectional sliding have not been analyzed. This work attempts to fill this gap. The aim of this work is to study the effects of normal load, frequency of oscillation, and temperature on the coefficient of friction of textured surfaces in lubricated reciprocating sliding and consequently to recommend pit area ratios for various operating conditions. The results of this study will allow in the future for the selection of multi-scale textured samples for tribological purposes, such as cylinder liner surfaces in internal combustion engines.

## 2. Materials and Methods

**Figure 1:**
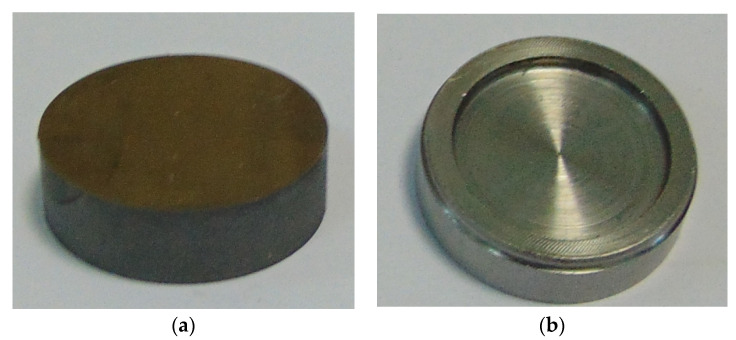
Untextured sample (**a**) and counter sample (**b**).

The experiments were carried out with reciprocating sliding using an Optimol SRV 5 tribotester equipped with a standard oscillation block in conformal lubricated contact. The contact zone had a ring shape as shown in Figure 1. The lower disc (sample) of 7.9 mm thickness had a diameter of 25 mm, while the upper disc (counter sample) of 5 mm thickness had a diameter of 18 mm. The lower and upper discs were made from 42CrMo4 steel of 44 ± 2 HRC hardness, which was achieved after heat treatment. The samples were either laser-textured or untextured, while the counter samples were untextured.

The surface roughness height of the untextured sample and of the textured samples in areas without dimples and of the counter sample, after grinding, determined by the Ra parameter, was 0.1–0.15 µm. Texturing was conducted using a SpeedMarker 300 laser engraver made by Trotec**^®^** equipped with a Ytterbium pulsed fiber laser FL 20 MOPA. Table 1 presents the parameters of laser texturing.

**Figure 2:**
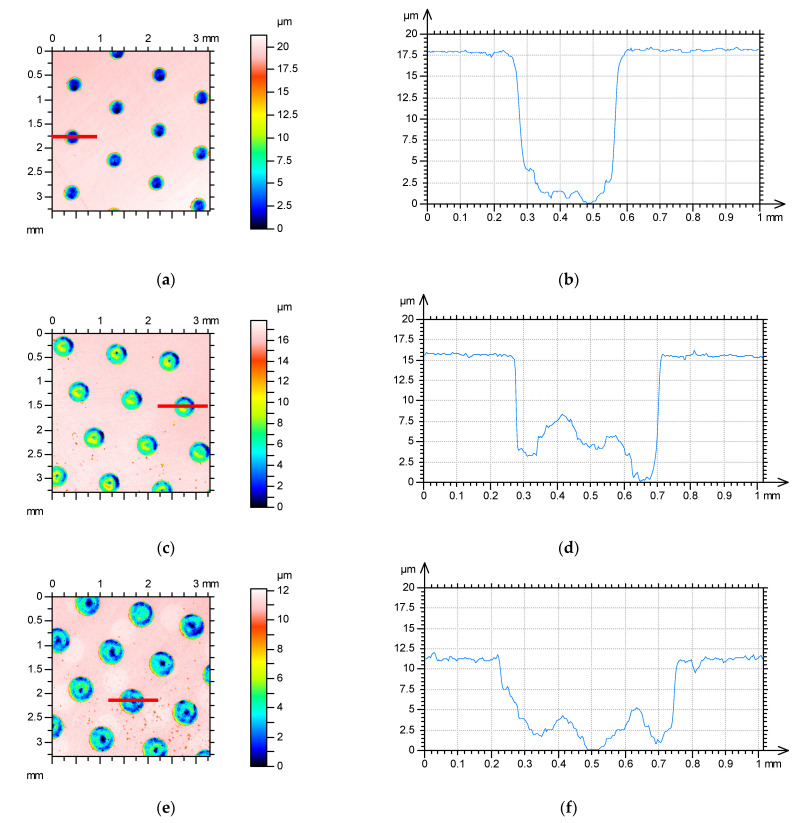
Contour plots (**a**,**c**,**e**) and extracted profiles (**b**,**d**,**f**) of textured disc surfaces for pit area ratios of 9% (**a**,**b**), 17% (**c**,**d**), and 25% (**e**,**f**).

Due to the highest popularity, the dimples had circular shapes. Textured surfaces with the following pit area ratios were tested: 9%, 17%, and 22%. The changes in the densities of the oil pockets were caused by changes in their diameters. The depths of the dimples were 15.5 ± 2 µm. The highest depth corresponded to the lowest pit area ratio. Figure 2 presents contour plots and selected profiles of textured surfaces.

**Figure 3:**
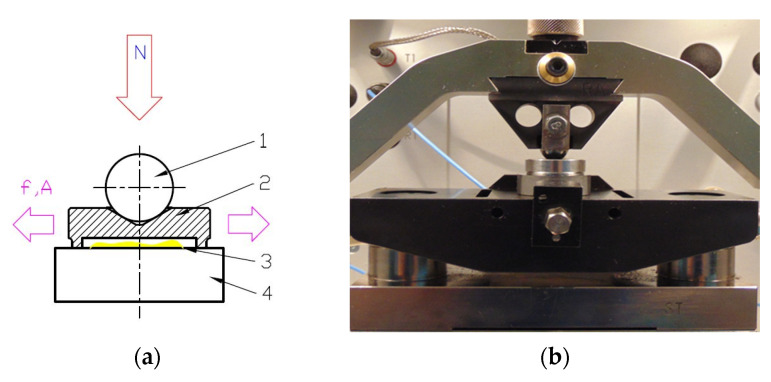
A scheme of contact configuration (**a**): N—normal load, f—frequency of oscillation, A—amplitude, 1—ball, 2—self aligning counter sample, 3—lubricant, 4—tested sample; picture of Optimol SRV5 test chamber with mounted samples (**b**).

In each experiment, the stroke was set to 3 mm. Experiments were carried out under various conditions: normal loads P were 40 and 80 N, oscillation frequencies f were 20 and 40 Hz, and temperature T values were 30 and 800 C. All combinations of operating parameters were performed, and for each sample, the number of subtests was 8. The duration of the subtest in each conditions was 5 min. The tests were carried out stepwise, starting from lower to higher temperatures. Figure 3 presents a schematic of the contact configuration and sliding condition of the testing. The low temperature period contained 4 subtests performed at small and high loads and low and high frequencies in all combinations. Between the low and high temperature periods, there was a cooling pause of 10 min, where load and frequency were set to 0. Such a break was necessary for temperature stabilization. The number of test repetitions was 3.

Before each test, two drops of L-AN-46 (approximately 0.08 ± 0.01 mL) mineral oil, which was also used in [38], were supplied to the inlet side of the test zone. It was machine mineral oil refined from selected oil distillates. Oil contains additives that prevent foam forming (kinematic viscosity at 40 °C: 43.2 mm^2^/s, ignition temperature 232 °C, solidifying temperature −24 °C, viscosity ISO classification: ISO VG 46). This lubricant was selected due to low amount of additives, because oil can lead to low friction just from additives themselves and consequently to misinterpretation of the obtained results. Before and after tests, surface topographies of the test samples were measured using a Talysurf CCI Lite white light interferometer. The measuring area of 3.29 mm × 3.29 mm contained 1024 × 1024 data points. Data were only leveled, and digital filtration was not used.

## 3. Results and Discussion

**Figure 4:**
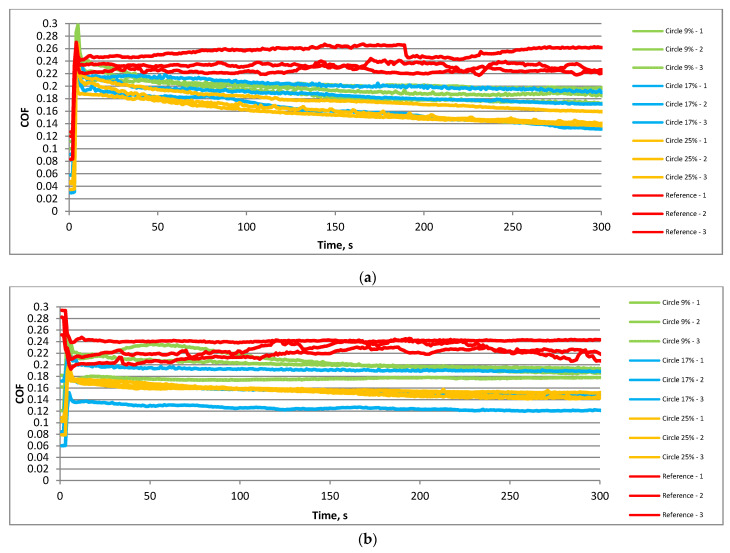
Courses of the coefficient of friction in the following operating conditions: f = 20 Hz; F = 40 N; T = 80 °C (**a**) and f = 20 Hz; F = 80 N; T = 80 °C (**b**).

Figure 4 presents the courses of the friction coefficient of the textured and untextured assemblies for the smaller frequency of 20 Hz and a higher temperature of 80 °C. When the normal load was smaller (40 N—Figure 4a), the highest coefficients of friction were obtained for the untextured sliding pair. Among assemblies containing textured samples, the smallest coefficient of friction was achieved for the highest pit area ratio, while it was the highest for the smallest density of dimples. Assemblies with textured samples with a middle dimple density were characterized by high variations of the friction coefficient between different test repetitions. The courses of the friction coefficient depended on the type of disc sample. For the untextured sample, the friction force was constant or increased as the test progressed. However, textured samples led to a decrease in the friction force with time due to disc surface texturing. This decrease occurred from the beginning of the test. An increase in the normal load to 80 N (Figure 4b) led to similar test results to those shown in Figure 4a. However, for textured sliding pairs, decreases of the friction force with time seemed to be smaller compared to tests with lower normal loads. In one test, the friction coefficient even increased with time for a textured sample of the smallest pit area ratio. The variation in the coefficient of friction was the smallest for the highest dimple density of disc samples.

**Figure 5:**
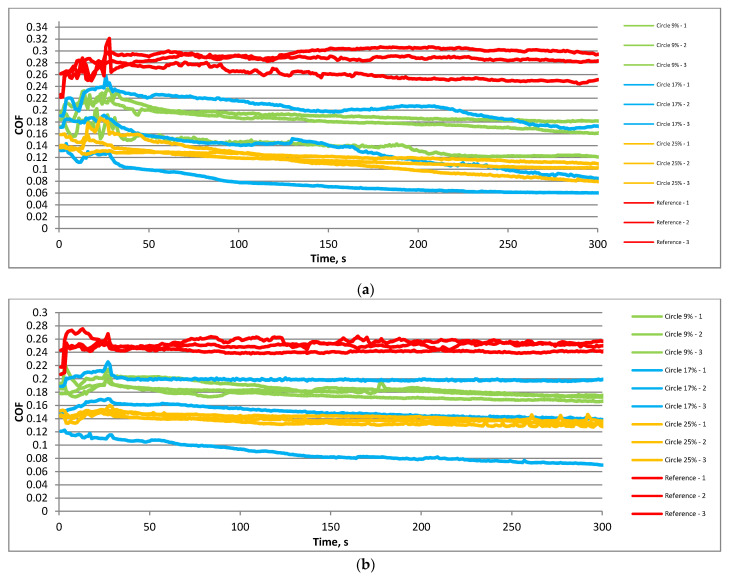
Courses of the coefficient of friction in the following operating conditions: f = 40 Hz; F = 40 N; T = 80 °C (**a**) and f = 40 Hz; F = 80 N; T = 80 °C (**b**).

Figure 5 presents the courses of the friction coefficient of the textured and untextured assemblies for the higher frequency of 40 Hz and the temperature of 80 °C. Similar to testing at a smaller frequency, untextured samples led to the highest coefficient of friction. Among textured samples, the smallest coefficient of friction was obtained for the highest pit area ratio, while the highest was obtained for the smallest dimple density, independently of the applied normal load. Among the textured assemblies, the variation of the friction force for various tests was the smallest for the highest pit area ratio, but the highest for the middle pit area ratio. Unlike tests at smaller frequencies, large fluctuations of the friction force occurred for the first 20 s due to an increase in the sliding speed; after this period, the coefficient of friction decreased for textured sliding pairs. The highest decrease occurred at a lower normal load. When the untextured specimens were tested, the coefficient of friction after initial large changes was typically constant as the test progressed.

**Figure 6:**
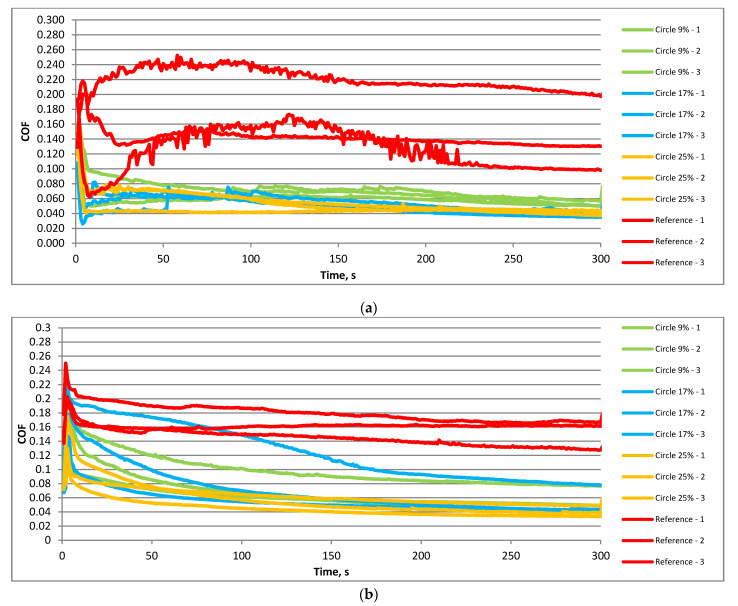
Courses of the coefficient of friction in the following operating conditions: f = 20 Hz; F = 40 N; T = 30 °C (**a**) and f = 20 Hz; F = 80 N; T = 30 °C (**b**).

Figure 6 presents the friction coefficient versus time for the smaller frequency of 20 Hz and the temperature of 30 °C. The highest variation and values of the friction coefficient were obtained for untextured samples when the load was lower (Figure 6a). When textured samples were tested, the coefficient of friction after initial fluctuations of about 100 s generally decreased. The final values between 0.04 and 0.06 were smaller compared to the tests carried out at higher temperatures. Among textured sliding pairs, the highest final values of the coefficient of friction were obtained for the lowest pit area ratios. An increase in the normal load to 80 N (Figure 6b) led to similar changes in textured and untextured sliding pairs with time. The coefficient of friction initially increased but after a few seconds decreased. This decrease was the lowest for the untextured sliding assembly. In this case, the final value of the coefficient of friction was the highest. Among the textured samples, the smallest fluctuations and values of the coefficient of friction were achieved for the highest density of dimples.

**Figure 7:**
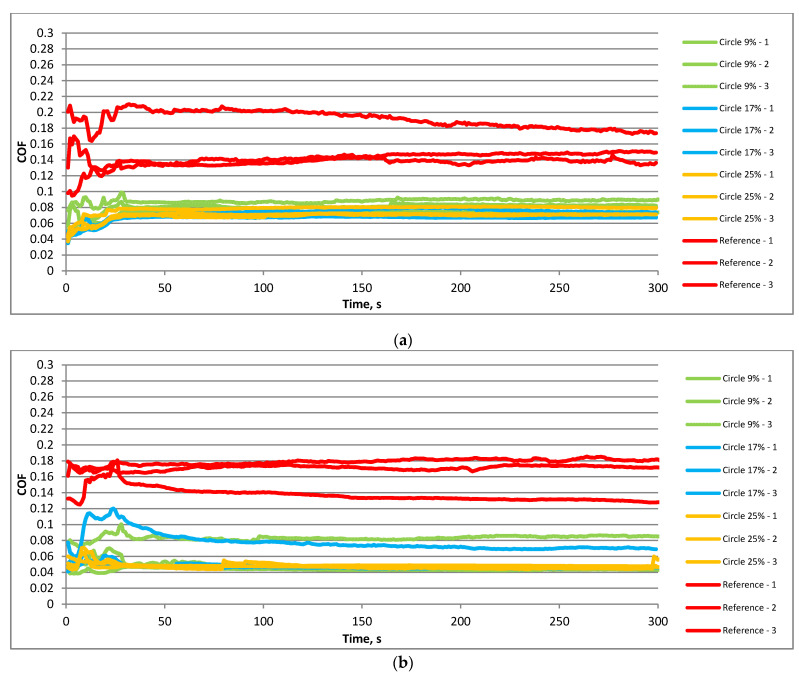
Courses of the coefficient of friction under the following operating conditions: f = 40 Hz; F = 40 N; T = 30 °C (**a**) and f = 40 Hz; F = 80 N; T = 30 °C (**b**).

Figure 7 shows the coefficient of friction versus time for textured and untextured assemblies under a higher frequency of oscillation of 40 Hz at a lower temperature of 30 °C. Similarly to working at higher temperatures, an increase in frequency led to an increase in duration of the initial fluctuation of about 30 s, independent of the normal load. For both normal loads, the highest value and the variation of the friction coefficient were obtained for an untextured sliding pair. When textured disc samples were tested, after initial fluctuations, the coefficient of friction was nearly constant. When the load was smaller, namely, 40 N (Figure 7a), all textured sliding pairs behaved similarly. For the higher load of 80 N (Figure 7b), the smallest variation and values of the coefficient of friction were achieved for the sliding pairs with the highest densities of dimples.

**Figure 8:**
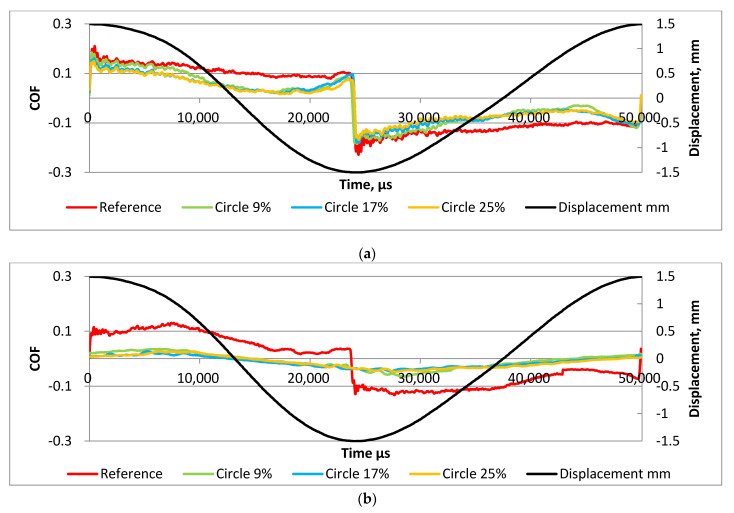
Coefficient of friction versus time within one stroke under the following operating conditions: f = 20 Hz; F = 40 N; T = 80 °C (**a**) and f = 20 Hz; F = 40 N; T = 30 °C (**b**).

The analysis of runs of the coefficient of friction in one stroke led us to similar findings. In both cases shown in Figure 8, the highest coefficients of friction were obtained for untextured samples. A decrease in temperature caused lower values of the friction coefficient, especially of textured assemblies. Among them, the highest coefficient of friction was obtained for the smallest pit area ratio of disc samples. The graphs shown in Figure 8 were obtained after stabilizations of the friction coefficients. The exact time of data extraction was 4 min 50 s after the start of the selected subtest.

**Figure 9:**
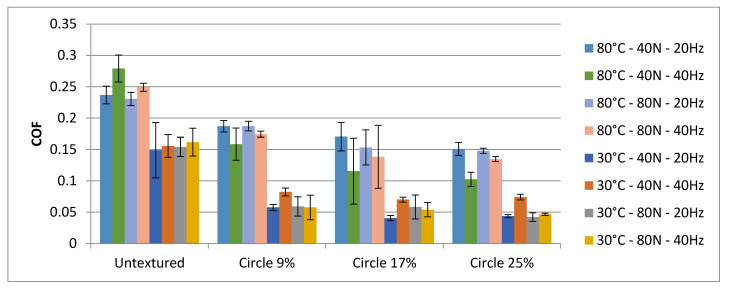
The mean values and error bars of the coefficient of friction of textured and untextured sliding pairs under various operating conditions.

Figure 9 presents the average values and error bars based on standard deviations of textured and untextured assemblies under various operating conditions. Higher coefficients of friction at higher temperatures compared to those obtained at lower temperatures are the main differences between the test results.

**Figure 10:**
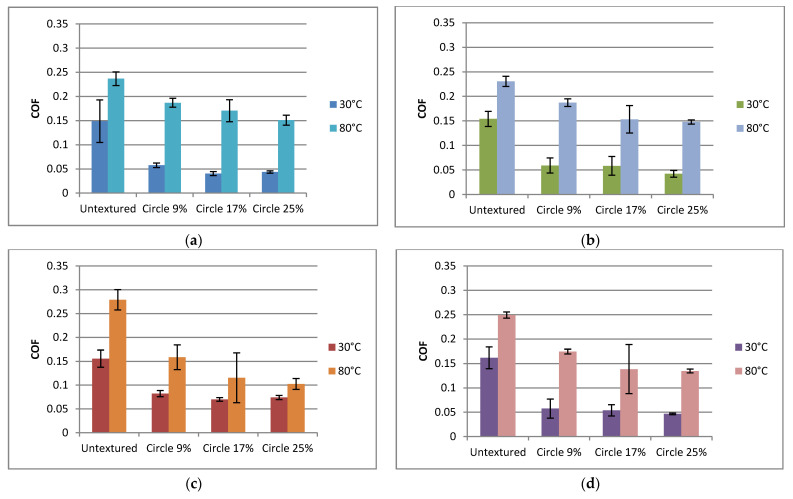
Average values and standard deviations of the coefficient of friction of textured and untextured sliding pairs for the following operating conditions: f = 20 Hz, p = 40 N (**a**); f = 20 Hz, *p* = 80 N (**b**); f = 40 Hz, *p* = 40 N (**c**); f = 40 Hz, *p* = 80 N (**d**).

To better characterize the effects of particular operating conditions on the tribological behavior of sliding pairs, additional analysis was necessary. Figure 10 presents the effect of temperature on the coefficient of friction of textured and untextured assemblies. In all analyzed cases, an increase in temperature led to an increase in the coefficient of friction. This increase was the smallest for untextured assemblies—1.45 times, on average—and was higher for larger frequencies of oscillation. For textured discs, an increase in the coefficient of friction due to a temperature rise was larger than that of the untextured sample, namely, 3.1 times, on average. In contrast to untextured sliding pairs, this increase was larger for smaller frequencies of oscillation. Surface texturing led to a reduction in the coefficient of friction. This reduction was greater at a lower temperature—3.1 times—than at a higher temperature—1.7 times—on average. The highest reduction in the coefficient of friction due to surface texturing was 4.6 times at a lower temperature and 2.5 times for a higher temperature. When the test was carried out at a higher temperature, the reduction was higher at a larger frequency of oscillation. At a lower temperature, the effect of pit area ratio on the friction coefficient was small; at a smaller frequency and normal load, the largest dimple density behaved better that the smallest one, and at a higher frequency of oscillation and a smaller normal force, the middle dimple density led to a smaller coefficient of friction than the smallest pit area ratio. However, at a higher temperature, the impact of density of dimples was visible—in all cases the highest density led to that smallest coefficient of friction compared to the smallest one. Vladescu et al. [28] also found that in more difficult operating conditions, the oil pockets should be wider. The middle density of dimples led to the highest variation of the friction coefficient. An increase in the friction coefficient as a result of the increase in temperature was caused by a decrease in the viscosity of the oil. Similar results were obtained in the reciprocal motion tests [22].

**Figure 11:**
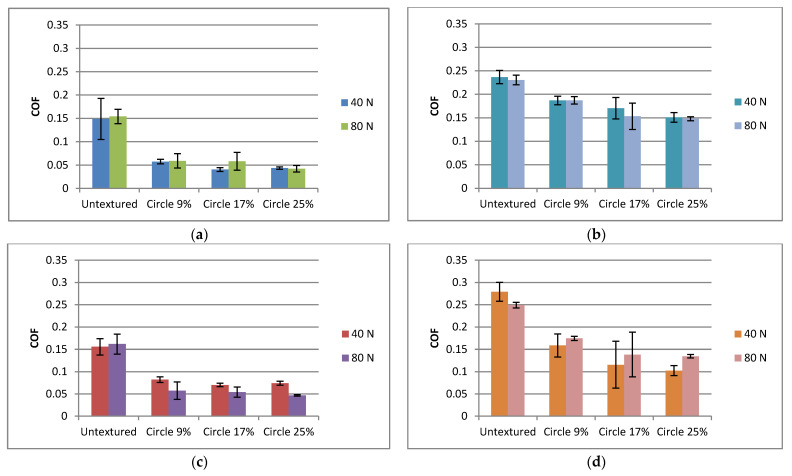
Average values and standard deviations of the coefficient of friction of textured and untextured sliding pairs for the following operating conditions: f = 20 Hz, T = 30 °C (**a**); f = 20 Hz, T = 80 °C (**b**); f = 40 Hz, T = 30 °C (**c**); f = 40 Hz, T = 80 °C (**d**).

Figure 11 presents the effect of the normal load on the coefficient of friction of the analyzed assemblies. When the frequency of oscillations was smaller (Figure 11a,b), the effect of the normal load on the coefficient of friction was marginal. The tendency was found that at lower temperatures, an increase in the normal load caused an increase in the coefficient of friction, with the opposite tendency occurring at higher temperatures. However, the effect of the normal load on the coefficient of friction was substantial when the frequency of oscillation was higher. When tests were carried out at a lower temperature (Figure 11c), an increase in load led to a decrease of the coefficient of friction, when textured sliding pairs were tested. This behavior can be explained by better accommodation of the contacted surfaces during the increase of the normal load. Similar performance was observed in reciprocal motion [43]. However, an opposing behavior was observed at higher temperatures (Figure 11d). When textured sliding pairs were tested, an increase in the normal load caused an increase in the coefficient of friction; the difference was substantial only for the highest pit area ratio of discs. An increase in temperature caused an increase in the coefficient of friction because the assembly operated in mixed lubrication. Perhaps when the resistance to motion was large (particularly near the dead center), better accommodation at higher load was not possible for textured assemblies. The shape of the Stribeck curve [9] may be helpful in explaining this behavior. Under mixed lubrication, an increase in normal load causes an increase in the coefficient of friction. On the contrary, an increase in the normal load of untextured sliding pairs caused a decrease in the coefficient of friction at higher temperatures and frequencies of oscillation. At lower temperatures, the effect of pit area ratio on the coefficient of friction was visible only for smaller normal load and frequency—the smallest density led to the largest friction. When the temperature was higher for both normal loads and frequencies of oscillation, the smallest pit area ratio caused higher friction than the largest density of dimples.

**Figure 12:**
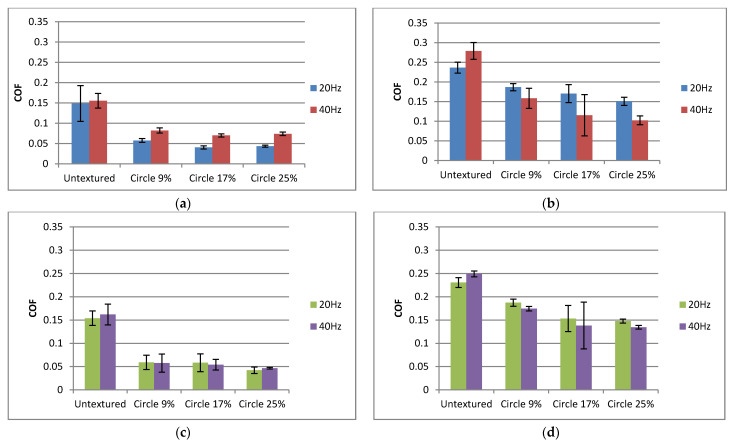
Average values and standard deviations of the coefficient of friction of textured and untextured sliding pairs for the following operating conditions: *p* = 40 N, T = 30 °C (**a**); *p* = 40 N, T = 80 °C (**b**); *p*= 80 N, T = 30 °C (**c**); *p* = 80 N, T = 80 °C (**d**).

Figure 12 presents the effect of the frequency of the oscillations on the coefficient of friction. When the normal load was smaller (Figure 12a), an increase in frequency (and sliding speed) caused an increase in the coefficient of friction. This behavior can be explained by the possibility of the presence of hydrodynamic lubrication at some places within the stroke; see Figure 8b. The opposing behavior occurred at higher temperatures (Figure 12b,d), when textured samples were tested, especially for the highest density of dimples on the surface of the disc. This behavior can be explained by the shape of the Stribeck curve [9]. Under mixed lubrication, an increase of speed led to a decrease of the coefficient of friction. However, the opposite behavior occurred for untextured assemblies. When the normal load was higher, and the temperature was lower (Figure 12c), the effect of the frequency of oscillations on the coefficient of friction was negligible. When the temperature was lower, the effect of pit area ratio on the friction coefficient was visible only for a smaller frequency and normal load—the smallest density of dimples led to the largest friction. At higher temperatures, the smallest oil pocket density caused a higher friction coefficient than the highest pit area ratio, independently of normal load and frequency of oscillation.

**Figure 13:**
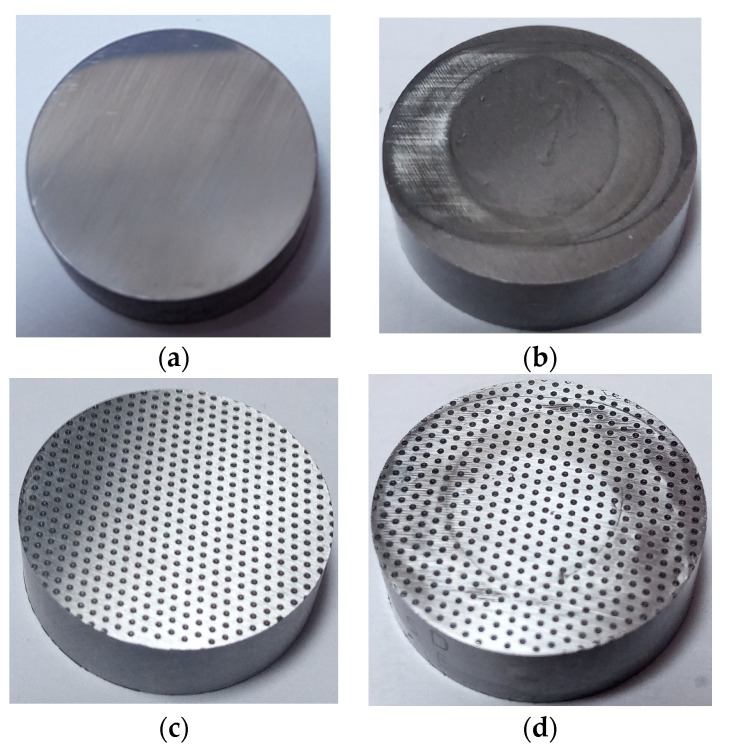
Photo of the untextured disc before the test (**a**), photo of untextured disc after the test with oil and wear products (**b**), photo of textured disc with pit area ratio of 25% before the test (**c**), and photo of textured disc with pit area ratio of 25% after the test with oil and wear products (**d**).

Figure 13 presents photos of selected discs before and after tests. Due to the similar hardness of the sample and counter-sample, the wear of the discs was marginal. Only the highest peaks/summits were truncated. The largest visible changes were observed on untextured discs (Figure 13b); however, surface topography changes were non-measurable. For wear analysis of this tribosystem, longer tests should be performed. Due to low wear with oil, the observations after tests did not give a clear view of the wear mechanism, because the oil was too clean (Figure 13b,d) and the amount of wear debris was small.

## 4. Conclusions

1. In all tested conditions, surface texturing led to friction reductions of sliding pairs in lubricated reciprocating motions. In most cases, the friction coefficient of textured assemblies decreased with time, contrary to behaviors of untextured sliding pairs.
2. Friction decreases were higher at lower temperatures (up to 4.5 times) compared to higher temperatures (up to 2.5 times).
3. The impact of the pit area ratio was visible at higher temperatures. The highest dimple density of 25% led to a smaller coefficient of friction than the smallest pit area ratio of 9%. The sliding pair with a middle density of 17% was characterized by large variation of the friction force. This behavior was obtained independently of normal load and frequency. At lower temperatures, the impact of the pit area ratio of a disc sample was visible only for smaller normal loads and frequencies of oscillation—the smallest density of dimples led to the largest friction.
4. At lower temperatures, the coefficients of friction were smaller compared to tests at higher temperatures.
5. For a higher frequency of oscillations at lower temperatures, an increase in normal load from 40 to 80 N led to a smaller coefficient of friction when textured surfaces were tested, contrary to work at higher temperatures. The opposite behavior was obtained for untextured samples. When the oscillation frequency was lower, the effect of the normal load on the coefficient of friction was marginal.
6. At lower temperatures and normal loads, a higher frequency of oscillation led to higher coefficients of friction of textured sliding pairs, contrary to tests at higher temperatures. When the normal load was higher, the higher oscillation frequency corresponded to a lower coefficient of the friction of textured assemblies.

## Figures and Tables

**Table 1:** Parameters of laser surface texturing.

Parameter	Value
Laser power	20 W (100%)
Focal length	254 mm
Focal diameter	64 µm
Pulse duration	1.5 ns
Pulse repetition rate	820 kHz
Marking speed	200 mm/s
Marking path pattern	Cross-line bidirectional

## Data Availability

Not applicable.
